# Implantation of Autologous Expanded Mesenchymal Stromal Cells in Hip Osteonecrosis through Percutaneous Forage: Evaluation of the Operative Technique

**DOI:** 10.3390/jcm10040743

**Published:** 2021-02-12

**Authors:** Enrique Gómez-Barrena, Norma G. Padilla-Eguiluz, on behalf of the REBORNE Consortium

**Affiliations:** 1 Cirugía Ortopédica y Traumatología, Hospital Universitario La Paz, IdiPaz, Universidad Autónoma de Madrid, 28046 Madrid, Spain; 2 Universidad Autónoma de Madrid, 28049 Madrid, Spain; norma.padilla@uam.es

**Keywords:** hip osteonecrosis, femoral head, percutaneous forage, surgical technique, cell therapy

## Abstract

Bone forage to treat early osteonecrosis of the femoral head (ONFH) has evolved as the channel to percutaneously deliver cell therapy into the femoral head. However, its efficacy is variable and the drivers towards higher efficacy are currently unknown. The aim of this study was to evaluate the forage technique and correlate it with the efficacy to heal ONFH in a multicentric, multinational clinical trial to implant autologous mesenchymal stromal cells expanded from bone marrow (BM-hMSCs). Methods: In the context of EudraCT 2012-002010-39, patients with small and medium-sized (mean volume = 13.3%, range: 5.4 to 32.2) ONFH stage II (Ficat, ARCO, Steinberg) C1 and C2 (Japanese Investigation Committee (JIC)) were treated with percutaneous forage and implantation of 140 million BM-hMSCs in a standardized manner. Postoperative hip radiographs (AP—anteroposterior and lateral), and MRI sections (coronal and transverse) were retrospectively evaluated in 22 patients to assess the femoral head drilling orientation in both planes, and its relation to the necrotic area. Results: Treatment efficacy was similar in C1 and C2 (coronal plane) and in anterior to posterior (transverse plane) osteonecrotic lesions. The drill crossed the sclerotic rim in all cases. The forage was placed slightly valgus, at 139.3 ± 8.4 grades (range, 125.5–159.3) with higher dispersion (*f* = 2.6; *p* = 0.034) than the anatomical cervicodiaphyseal angle. Bonferroni’s correlation between both angles was 0.50 (*p* = 0.028). More failures were seen with a varus drill positioning, aiming at the central area of the femoral head, outside the weight-bearing area (WBA) (*p* = 0.049). In the transverse plane, the anterior positioning of the drill did not result in better outcomes (*p* = 0.477). Conclusion: The forage drilling to deliver cells should be positioned within the WBA in the coronal plane, avoiding varus positioning, and central to anterior in the transverse plane. The efficacy of delivered MSCs to regenerate bone in ONFH could be influenced by the drilling direction. Standardization of this surgical technique is desirable.

## 1. Introduction

Treatments for osteonecrosis of the femoral head (ONFH) are still a matter of considerable debate. Non-operative treatments have been associated with high radiographic failure rates (at a mean of 72%) consistently throughout the years [1]. Due to a high degree of heterogeneity across various studies, best individual stage-dependent treatment options and especially the correct indications for surgical treatment are largely unknown. Treatment with forage drilling, the so-called core decompression (CD), is the classic joint-preserving alternative to treat early cases of ONFH, initially proposed to decrease the intraosseous pressure in avascular osteonecrosis of the femoral head [2]. However, the results of CD to avoid femoral head collapse and eventual total hip replacement (THR) are highly variable. In a systematic review, radiological progression after forage averaged 44% of treated cases in studies done before 1992, with an improvement to 37% failure in more recent studies [1]. The preoperative radiographic stage [3] or the extent and location of the osteonecrotic lesion have been related to the failure of forage treatment [4]. Particularly, CD treatment of stage III and beyond is associated with up to 100% failure (radiographic progression or THR conversion) [3,4,5]. Large necrotic lesions and osteonecrosis extension laterally to the acetabulum edge (the so-called C2 lesions according to Sugano et al. [6]) caused femoral head collapse even in asymptomatic hips without treatment [7]. The CD failure rate was found to be higher in hips with medium and large lesions (more than 15% estimated volume), and in hips with more lateral lesions, rather than medial or central [4].

The forage or CD technique has substantially evolved given the limited, variable efficacy. Different technical proposals include multiple small drilling [8,9,10,11,12] versus a single larger diameter [8,13], with the risk of occasional fractures [11]. Regarding the positioning of the drill, not only fluoroscopy but also computer-guided [14] and even magnetic resonance (MR) guidance techniques [15] have been proposed, with claims that up to 100% reach the target (the osteonecrotic lesion). Direct vision of the drilled tunnel through endoscopy [16] and even hip arthroscopy [17] have been used to assist CD with a tunnel or intra-articular visualization. Other modifications include the incorporation of different augmentation grafts or substitutes, such as calcium phosphate and sulfate [18], demineralized bone matrix [19], grafts with bone morphogenetic protein (BMP) [20] and various combinations, as recently reviewed [21].

Considerable interest has been generated by the advances in cell therapy to regenerate bone, and Hernigou early on confirmed the benefits of bone marrow (BM) grafting injected through the forage [22]. The use of cell therapy approaches has increased since then, whether in the bone marrow concentration (BMC) or after cell expansion, aiming to deliver sufficient numbers of mesenchymal stromal cells (MSCs) [23] and offering a significant improvement over CD alone [24]. Yet, some potential patient-related aspects may affect the outcome, particularly considering the different etiological and possibly pathophysiological aspects within the ONFH. Of note, autologous treatments such as many cell therapy strategies may also impact on the therapy results, and therefore treatment standardization is paramount.

Efficacy may not only depend on patient- and disease-specific aspects (stage, volume, location of the necrosis, acute or chronic phase), but also on technical specificities that are poorly understood. While surgeons usually aim to perform the forage drilling towards the lesion, the cell distribution and subsequent efficacy may also be modulated by this drilling. Among the uncertain issues, some are technical, such as drill diameter, single versus multiple drilling or the location and direction of the drilling related to the lesion, towards the central area of the femoral head or the weight-bearing area (WBA). In preclinical models, the biodistribution of MSCs was proven to remain within the injected femoral head [25], confirming the tropism of injected BM-derived MSCs for the bone surface. Despite this finding, the reach of cells may vary depending on the drill location, and thus affect the efficacy. We hypothesise that the surgical technique, and particularly the drill location, may affect the treatment outcome.

At this point, the study aim is to evaluate the variability of the forage positioning and to identify how this variability may affect the efficacy of cell therapy, framed in a clinical trial injecting expanded autologous BM-derived MSCs in the femoral head with osteonecrosis Ficat–Arlet or ARCO stage II. To meet the aim of the study, we evaluated the efficacy of the technique related to the location of the osteonecrosis and the location of the forage tunnel.

## 2. Materials and Methods

Anonymized imaging from 22 patients treated for osteonecrosis of the femoral head under the Ortho 2 clinical trial (EudraCT 2012-002010-39) in the REBORNE EU-funded project (Regenerating bone defects using new biomedical engineering approaches, FP7 HEALTH-2009-1.4-2, Grant Agreement 241879) is the material under study [26]. Briefly, the trial was conducted in five clinical centers from four European countries (France, Germany, Italy and Spain) from March 2014 to June 2015. Patients were treated with percutaneous forage plus implantation of 140 million expanded mesenchymal stem cells (clinical grade) from bone marrow in a single injection of up to 7 mL of albumin (dose of 20 x 10^6^ cells/mL). All included patients agreed to their participation and signed an appropriate informed consent form (Ethics Committee authorization code, coordinating center: HULP 3875). Patients were 19 males and 3 females with a mean ± sd (range) age of 43 ± 10 (21-62) years and a mean ± sd (range) time since initial diagnosis of 2.3±2.2 (0.1–7.6) months. The ONFH was idiopathic (40%) or due to corticosteroid treatment (25%) or other non-traumatic causes (35%). All cases were classified as stage II by Arlet and Ficat [2,27], Steinberg [28] or ARCO [29], as all these classifications converge on this stage, even after very recent modifications [30]. The volume of the necrosis [31], as a percentage of the sphere circumscribing the femoral head, was estimated (mean = 13.3%, range: 5.4% to 32.2%). The Japanese Investigation Committee (JIC) on osteonecrosis staging [6] was considered to evaluate the location of the lesion.

**Figure 1:**
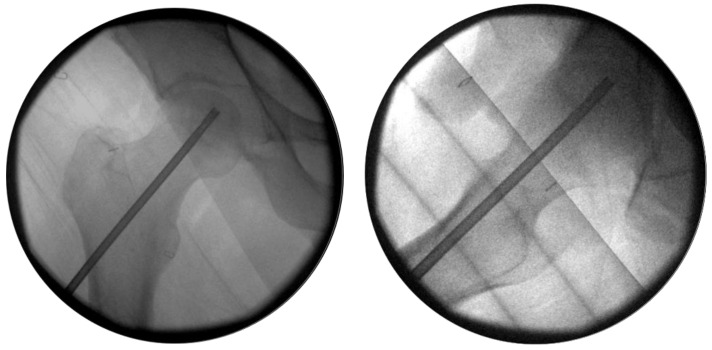
Intraoperative fluoroscopic images showing the drilling tunnel for implantation of cell therapy in anteroposterior (AP) (left) and lateral (right) plane.

During surgery, antimicrobial prophylaxis and analgesia were performed as per local protocol. After anesthesia (general 68%, spinal 32%), patients were positioned supine on a fracture table. A radiological C-arm was placed and both anteroposterior (AP) and axial views of the femoral neck and head were checked under fluoroscopy. Following a minimally invasive approach, with a 1 cm incision laterally to the proximal femur, a guide wire was drilled from the lateral cortex of the subtrochanteric femur into the femoral head lesion, under fluoroscopic AP and axial control. Then, a 4 mm cannulated drill was introduced along the guide wire into the femoral head (Figure 1), guided by intraoperative fluoroscopy. Per protocol, one syringe was used to inject 7 mL within the forage tunnel in one single administration, slowly progressing to avoid overpressure (about 2 min were required to complete the injection). The guide wire was reintroduced to facilitate the clearing of the cannulated drill, and after 2–3 more minutes, the drill was removed without leakage. No sealing was required. The injected cell product consisted of a dose of 140 million MSCs suspended in 5% human albumin (20 million MSCs/mL). Cell expansion details have been published elsewhere [26,32]. Each patient underwent repeated radiographs (at 6 weeks, and 3, 6, and 12 months) and magnetic resonance imaging (MR at 3 and 6 months) during the clinical trial. The final evaluation of efficacy was completed after a minimum of 5 years’ follow-up.

**Figure 2:**
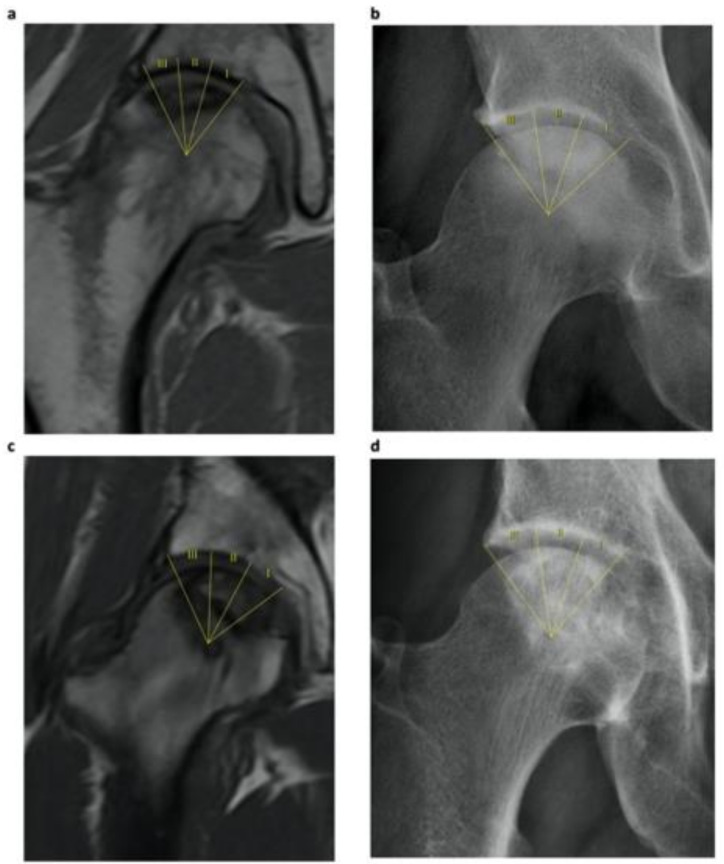
Coronal location of the lesion in the weight-bearing area through the 2001 system, comparing T1-coronal MR with AP radiograph of two cases. In case 105, lesion (**a**) was classified as C2 as it occupied three thirds of the weight-bearing area and extended laterally to the acetabular edge; while lesion (**b**) was classified as C1, not extending further than the acetabular edge. In case 202, lesions through both imaging techniques in (**c**) and (**d**) were classified as C1. I, II, III: Weight-bearing area thirds

To determine the coronal location of the necrotic lesion, we used the 2001 classification system, proposed by the Japanese Investigation Committee [6], on preoperative T1-MR and AP X-rays. The classification scheme consists in classifying the lesion in the weight-bearing area (WBA) as one of four types: A, B, C1 and C2, based on the central section of the femoral head on T1-weighted coronal MR or the AP radiograph (the image to evaluate was the coronal section when the anterior trochanter appears). Type A lesions occupied up to the medial third of the WBA. Type B lesions occupied up to the medial two-thirds of the WBA. Type C was divided into C1, occupying more than the medial two-thirds of the WBA and not extending laterally to the acetabular rim, and C2, occupying more than the medial two-thirds of the WBA and extending laterally to the acetabular rim. Figure 2 shows the system used in two different cases.

**Figure 3:**
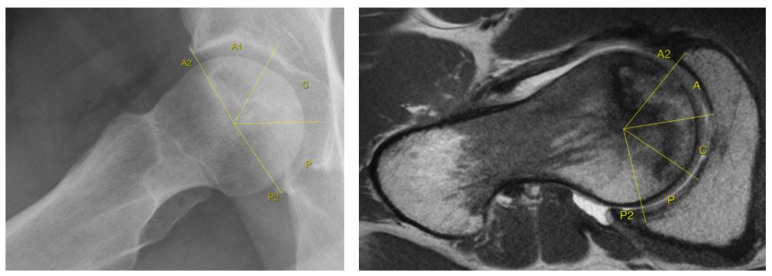
Location of the osteonecrosis through the anterior, central, posterior (ACP) method (from/to: anterior, central, posterior) comparing two images of case 101 in which (left) the lateral radiograph was classified as ACP and (right) the axial T1-MRI plane was classified as A2CP. The value of anterior 2 or posterior 2 means that the lesion surpasses the acetabular anterior edge (A2) or the posterior acetabular edge (P2).

To estimate the transverse lesion location from anterior to posterior, we defined a so-called anterior/central/posterior (ACP) method on preoperative T1-transverse MR sections and lateral or axial radiographs of the hip. The method consists in identifying the extension of the lesion in three regions of the femoral head (from/to: anterior/central/posterior), taking as a reference the osseous acetabular rim (from the anterior to the posterior edge). The value of anterior 2 (A2) or posterior 2 (P2) means that the lesion surpasses the anterior acetabular edge (A2) or the posterior acetabular edge (P2). To evaluate the transverse plane in MR sections, we set the height in the section where the anterior trochanter appears, using the comparative function of OsirixMD software v9.1 (Pixmeo SARL; Geneva, Switzerland) to help us adjust the coronal view. We use a radial angle circle tool to divide the region into three zones. Figure 3 shows an example.

**Figure 4:**
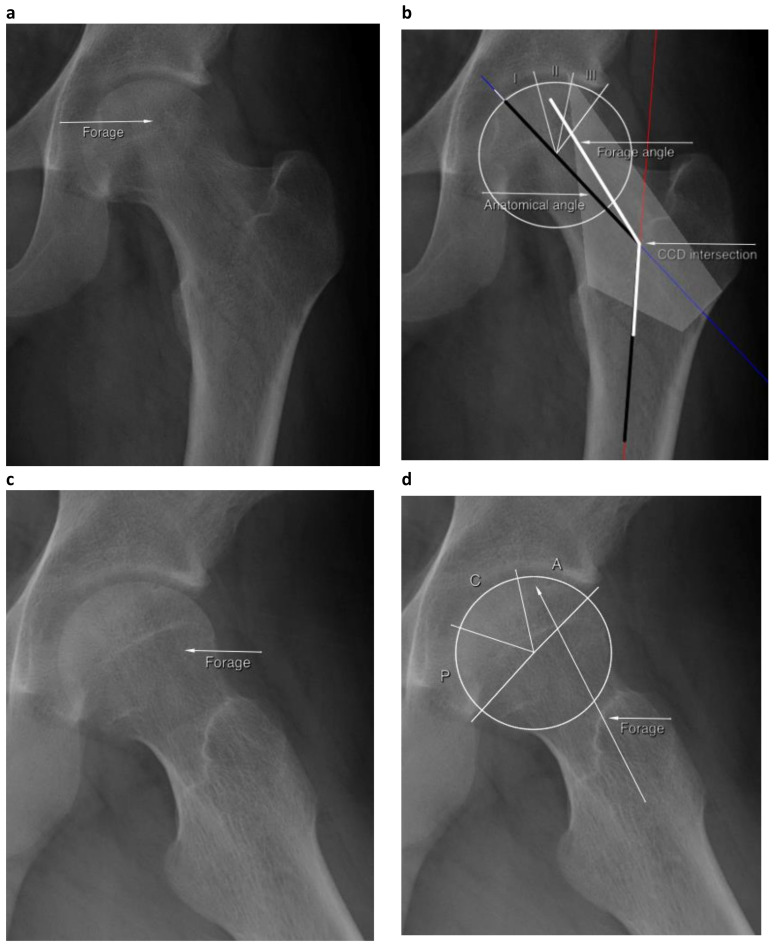
Forage location in postoperative radiographs at 1.5 months after surgery, case 103, showing (**a**) forage drilling in AP radiograph, and (**b**) the radiological measurements of the anatomical angle, forage angle and forage location in the second third of the weight-bearing area (WBA), using the Japanese Investigation Committee (JIC) 2001 staging. In (**c**), forage drilling in lateral radiograph, and (**d**) location of the forage in the anterior section of the femoral head, using the ACP method.

Finally, postoperative (1.5 or 3 months since surgery) anteroposterior radiographs were examined to measure the anatomical angle and the forage angle, taking as a reference the intersection point of the cervicodiaphyseal angle (caput–collum–diaphyseal angle, CCD), and to locate the forage tunnel in the WBA thirds (I, II, III), as seen in Figure 4a,b. Postoperative lateral or axial radiographs of the hip were used to classify the forage by the ACP method (Figure 4c,d). Both planes were analyzed to verify if the forage crossed the sclerotic rim into the necrotic lesion.

All images were processed, measured, and classified with OsirixMD software [33]. Mean and standard deviation for continuous variables and the percentage for categorical variables were reported. For analytical analysis, we used Stata statistical software, release 12 (StataCorp LP; College Station, TX, USA). A percent of agreement test (kappa test) was conducted to compare the osteonecrosis classification (by the 2001 system and ACP method) between MR and radiographic images. The degree to which both measurements were equivalent (agreement) was considered slight (if kappa was from 0–0.20), fair (from 0.21–0.40), moderate (from 0.41–0.60), substantial (from 0.61–0.80) or almost perfect (if >0.80) [34]. The dependent variable for the analysis was the proportion of healed/non-healed cases. Comparisons of the lesion classification, location and forage were conducted using MRI. Mean differences and variance were reported using adequate parametric or non-parametric tests. Fisher’s exact test was used for proportion comparisons. The Kaplan–Meier survival function and log-rank tests were used to evaluate the equality of failure rates across groups.

## 3. Results

### 3.1. Treatment Efficacy and Extension of the Lesion

Characteristics of the osteonecrosis lesion and the forage technique are listed in Table 1, by study case. ONFH lesions were classified following the abovementioned JIC 2001 staging system [6] related to the WBA, with C1 in 8/22 cases (36%) and C2 in 14/22 cases (63%) using the T1-coronal MR sections, and C1 in 11/22 cases (50%) and C2 in the remaining 11 cases using AP X-rays. Inconsistencies between radiographs and MR were found in 9/22 cases (41%). The percentage of agreement between MRI and X-rays in the classification was considered moderate at 60% (CI, 95%: 0.37–0.81; *p* = 0.001). There was no difference in efficacy related to the JIC extension of the lesion in the coronal plane, whether the extension was evaluated in radiographs (Fisher’s exact test ji^2^ = 2.3; *p* = 0.311) or in MRI (Fisher’s exact ji^2^ = 0.8; *p* = 0.613). Therefore, the coronal extension of the lesion was not associated with an increase in the treatment failure after the delivery of autologous, expanded BM-MSCs (log-rank test ji^2^ = 0.7; *p* = 0.397).

The location of the osteonecrosis by the ACP method in MR studies was defined as A2CP in 11/22 cases (50%), ACP in 6/22 cases (27%) and A2C in 5/22 cases (23%). Using lateral or axial radiographs, the location was defined as ACP in 13/22 cases (59%) and A2CP in 9/22 cases (41%). The inconsistency between both types of images was 50% (11/22 cases). The percentage of classification agreement between MRI and radiographs in our cases was considered slight, 18% (CI, 95%: 0.01–0.35; *p* = 0.042). Then, the transverse evaluation was performed on MRI for the final analysis. There was no difference in the efficacy (bone healing) by the type of ACP lesion in MRI (Fisher’s exact ji^2^ = 2.5; *p* = 0.314). Therefore, the transverse extension of the lesion was not associated in this series with an increase in the treatment failure after the delivery of autologous, expanded BM-MSCs (log-rank test ji^2^ = 2.2; *p* = 0.327).

### 3.2. Treatment Efficacy and Forage Location

The mean anatomical angle of the proximal femur in grades was 133.7 ± 5.2 (range, 125.8–142.4), the mean intermediate angle forming the WBA-I (as per Figure 2) was 142.9 ± 4.7 grades (range, 133.7–151.8) and the mean intermediate angle forming the WBA-II was 152.2 ± 4.7 grades (range, 141.4–163.2). On average, the forage was placed with an angle of 139.3 ± 8.4 grades (range, 125.5–159.3). The variance ratio test between the anatomical angle and the forage angle reached statistical significance (*f* = 0.36; *p* = 0.018), with a Bonferroni correlation of 0.5 (*p* = 0.028).

**Figure 5:**
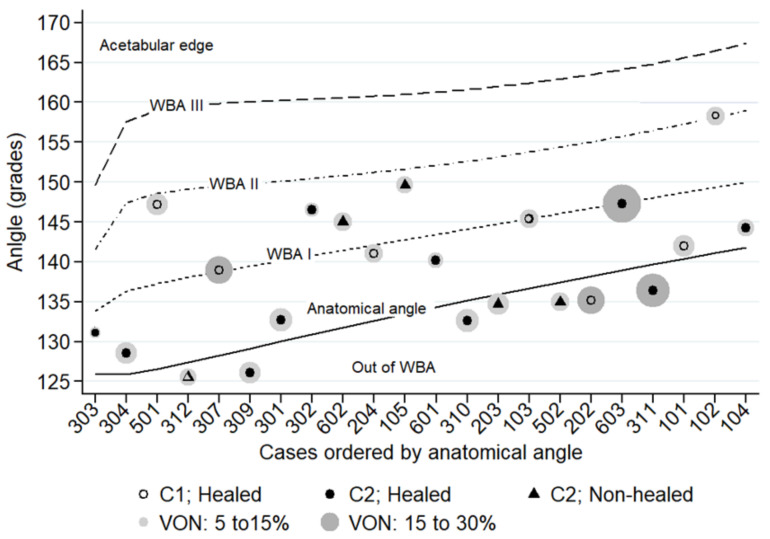
Distribution of the forage in the weight-bearing area, as per JIC 2001 scheme. C1: lesion occupying the three thirds of the WBA; C2: lesion occupying the three thirds of the WBA and extending laterally to the acetabular edge; VON: volume of osteonecrosis as a percentage of the femoral head (for location of the WBA, please see Figure 2).

The drilling crossed the sclerotic rim in all cases. Seven forages were placed inside WBA-I (32%), eight inside WBA-II (36%) and seven (32%) outside the WBA. The failure rate for cases with forage outside of the WBA (all in a varus position compared to the anatomic cervicodiaphyseal angle) was 42.8% (3/7) versus 13.3% (2/15) in forages placed within the WBA. In this sense, the efficacy of the injected cells to heal the lesion (in terms of avoiding osteonecrosis progression and/or THR conversion) was significantly higher when the forage was performed in the weight-bearing area (log-rank test ji^2^ = 3.85; *p* = 0.049). There was no significant difference in the failure rate (ON progression and/or THR conversion) when the forage was performed within the 1st or the 2nd portion of the femoral head weight-bearing area (see Figure 5, WBA-I and WBA-II) (log-rank ji^2^ = 1.7; *p* = 0.280).

**Figure 6:**
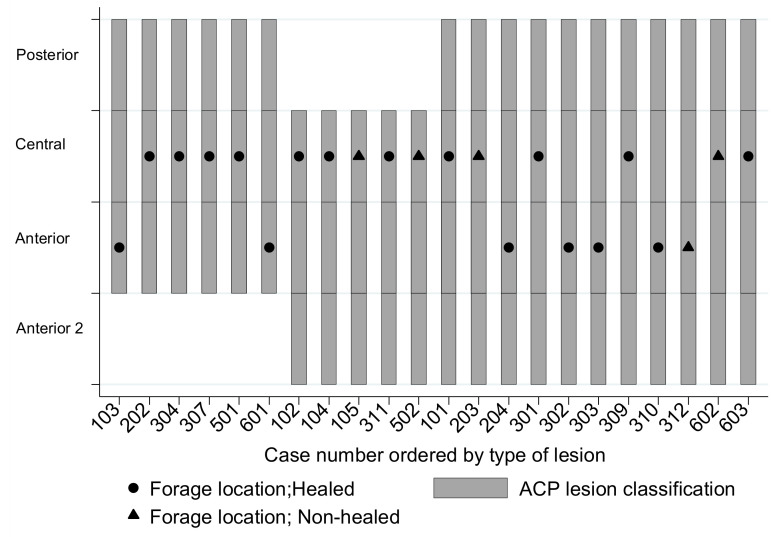
Case by case transverse location of the forage in the osteonecrotic (ON) lesion from anterior to posterior (ACP method described in Figure 3 and Figure 4d).

The transverse location of the forage (ACP) in the postoperative MR sections was central in 68% of cases (15/22) and anterior in the remaining 32% (7/19) of cases (Figure 6). The ACP location of the forage drill was not associated with bone healing (Fisher’s exact test ji^2^ = 0.41; *p* = 0.477). The failure rate of cases with anterior forage was 14% (1/7) and 27% (4/15) with central forage (Figure 6), with no statistically significant difference (log-rank test ji^2^ = 0.17; *p* = 0.681). No differences in bone healing were found between the anterior or central location of forage and the anterior extension of the lesion (Fisher’s exact test ji^2^ = 0.42; *p* = 0.999).

The mean preoperative volume of osteonecrosis was 13.4 ± 5.9 % (range: 5.4–32.2), as a percentage of the spherical model of the femoral head and did not influence the healing in this homogenous series. No differences were seen in the preoperative volume between the healed and the non-healed cases (Mann–Whitney test *p* = 0.514) and no differences were seen related to the coronal ONFH location, as per the JIC 2001 classification (Mann–Whitney test *p* = 0.185), or the transverse ONFH location, as per the defined ACP method (Kruskal–Wallis test *p* = 0.709). When comparing cases with small ONFH lesions (volume under 15%) to those with medium-sized lesions (over 15%), the failure rate was not statistically different (log-rank test ji^2^ = 1.7; *p* = 0.19). Adjusting the category of the small or medium preoperative volume of ONFH by the ACP location, no differences were observed in the failure rate (log-rank test ji^2^ = 1.2; *p* = 0.277), not even when adjusting with the JIC 2001 (A, B, C1, C2) type of lesion (log-rank test ji^2^ = 2.6; *p* = 0.451).

## 4. Discussion

Although non-surgical treatments may be an option in this type of patient, the surgical technique with regard to the position of the forage to deliver cell therapy was investigated in this study. The relevance of this issue is that cell therapy may regenerate bone within the osteonecrotic femoral head, but its distribution is unclear and therefore the efficacy may be affected by the way these cells are delivered. The main finding was the association of treatment failure with more varus forage positioning in the coronal plane, close to but outside the weight-bearing area of the femoral head. In the transverse plane, we could not find an association with either the anterior or central location of the drilling.

The technique’s efficacy may be related to the lesion. The influence of the lesion location in the treatment of ONFH has long been debated. After the original and extended Ficat and Arlet staging, Steinberg (later, University of Pennsylvania classification) [28], the *Association pour la Recherche de la Circulation Osseuse or Association Research Circulation Osseous* (ARCO) and the *Japanese Investigation Committee* (JIC) offered different approaches to expand the ONFH classification and integrate the prognostic value of extended locations of the lesion. Even if a recent Delphi approach to the ARCO classification contradicts this view [30] and does not include the subdivision according to the size/location/length of the necrotic area, the use of the JIC classification recommends surgery in type C2 lesions in a large series due to the increased risk of collapse [35]. In our study, we limited our inclusions to stage II cases (X-ray abnormal, MRI abnormal, changes seen in the femoral head, no evidence of subchondral fracture, fracture in the necrotic portion or flattening of the femoral head), and this stage is stable across all classifications. However, in a homogenous, controlled series, we did not find differences between healed and non-healed cases, adjusting for the JIC C1 and C2 distribution. We can then conclude that the expanded MSCs delivered in this study seem to equally heal all lesions extending into the coronal plane. This includes C2 lesions, which are more prone to femoral head collapse, as shown by other authors [35].

Besides this debate, the imaging evaluation to classify the lesion may also be a problem, as the assessment of the Ficat–Arlet and ARCO staging concluded that these classifications offer poor interobserver reliability and fair intraobserver variability [36]. We could observe that the coronal evaluation of the lesion location in radiographs and MR was in moderate agreement, and in neither of those evaluations was the efficacy associated with the location of the lesion.

We also defined anterior, central and posterior areas of the femoral head as a way to understand the transverse extension of the lesion, in the philosophy of the JIC classification for the coronal plane [6]. The need for this transverse description of the lesion was due to the planned analysis of the forage positioning not only in the coronal plane but also in this transverse plane. We observed that the agreement between the lesions’ transverse location on radiographs and MRI sections was only slight, possibly due to the variability in the radiograph positioning for lateral or axial hip projections. This being the case, our analysis was performed on MR transverse sections. No clear changes in efficacy were seen whether the lesion was more or less anterior in the femoral head, and we therefore conclude that even anterior lesions can be cured with the proposed technique.

The forage technique was then considered when related to treatment efficacy. The forage or CD technique has varied substantially in decreasing the potential risks, such as fracture [3] secondary to drilling or impaired biomechanical competence of the proximal femur [37]. In our case, we defined one single drilling of 4 mm in diameter and experienced no mechanical complications. The introduction of grafts often requires larger drilling, up to 9 mm with expandable reamers [38], the so-called advanced core decompression to remove the necrotic tissue [18]. The introduction of grafts has been claimed to be superior to standard CD procedures in a large series at 10 years when adjusted for Ficat stage [39], even if the clinical relevance is limited (58.1 vs. 57.9% 10-year survivorship). However, the size of the lesion may be a determinant when selecting this more aggressive technique. Small lesions (under 15% head volume) required 7% THR conversion, while medium and large lesions (over 15%) required 31 and 33% THR conversion after CD plus graft [31], and therefore, the volume was associated with the prognosis. This view has been further supported by new MR techniques [40]. In our study, most of the lesions were medium sized and no clear association was seen with treatment failure. The still unclear mechanism of action of this medicinal product may justify variable amounts of bone regeneration. This fact may be at the origin of different treatment outcomes and will deserve further studies.

The positioning of the forage has not been previously investigated. Furthermore, when multiple drilling (3 mm) is planned instead of a single, larger drilling [10], its positioning becomes even more unpredictable. The aim of reaching the ischemic area [14] is well established, with the help of fluoroscopy or other techniques, but when the lesion extends both in the coronal and transverse planes, the advisable place to deliver cells is unclear. We understood that crossing the sclerotic rim was necessary, and this condition was fulfilled in all the studied procedures. However, it is unclear how the infused cell therapy may be distributed in the femoral head. In experimental drilling and MSC injection in pig femoral heads [25], cells remained confined at the site of injection, attached to the bone trabeculae. Therefore, the positioning of the drill may affect the regenerative potential of the delivered cell therapy. Within the osteonecrotic lesion (particularly if widely extended) and to foster the bone regeneration proximal to the sclerotic rim, the surgeon may aim at the weight-bearing area in a more valgus drill positioning, or else aim at the central–central or even central–inferior area (such as in the fracture fixation techniques) in a more varus drill. We investigated the drill positioning related to the WBA and found that the regeneration obtained with a more varus drilling (under the anatomical CCD angle of the proximal femur) was less efficacious in the avoidance of failure. This finding recommends delivering cell therapy within the WBA in the coronal plane, with a valgus orientation of the drill. No differences were seen between weight-bearing areas I or II, and therefore, placing the drilling too valgus may not be required to improve the results. In the transverse plane, the more central or anterior drilling did not provide an advantage to bone regeneration. However, the postoperatively evaluated variability of the drilling was found to be moderate. A clinical protocol with a precise drilling angle (compared to the patient anatomical angle) may decrease this variability and help the surgeon to make intraoperative decisions regarding the surgical technique of the forage.

This study is based on postoperative imaging after an early clinical trial, and the number of cases is limited. This is a major limitation of the study, as occurs with many reports on osteonecrosis treatment. However, the fact that it is based on a precise protocol for including cases (specifically, stage II, symptomatic, early cases), performing the technique, delivering a standardized advanced treatment (such as 140 million autologous expanded MSCs) and following patients is also a study strength that has helped us to homogenize the results. Other limitations include the potential influence of different diagnoses, the variable regenerative potential of autologous cells from each patient, the unknown spatial proliferation of delivered cells and the evolution of these cells within the necrotic tissue. All these limitations due to the biology of the treatment may influence the surgical efficacy of the procedure. This bone regeneration is still poorly understood within the osteonecrotic lesion and will require further studies. Still, we believe that the role of adequately positioning the cells within the femoral head during the surgical procedure needs to be appraised and standardized, to avoid an important source of potential variability in the current treatment of early stages of osteonecrosis by means of cell therapy.

## 5. Conclusions

The drilling orientation of the percutaneous forage in the coronal plane within the weight-bearing area of the femoral head, slightly valgus compared with the anatomical CCD angle, increased the efficacy of bone regeneration when delivering cell therapy to the osteonecrotic femoral head in this study. In the transverse plane, central or anterior drilling were similarly efficacious. The drilling orientation should be standardized in a clinical protocol to percutaneously treat femoral head osteonecrosis with cell therapy, considering the injury location and spread, because the efficacy of the cells delivered in the treatment may be influenced by the surgical procedure.

## Figures and Tables

**Table 1:** Classification of the osteonecrosis and forage based on the location of the lesion in the weight-bearing area (coronal) and on the ACP method (transverse).

Case Number	Side	ON Location (Coronal, JIC) *		ON Location (Transverse, ACP) **		Forage Location (WBA Thirds) ^†^	Forage Location (ACP)
		*MR-T1*	*X-ray AP*	*MR-T1 Transv/Cor*	*X-ray Lat.*		
101	Right	C2	C1	A2CP	ACP	I	Central
102	Right	C1	C2	A2C	ACP	II	Central
103	Left	C1	C2	ACP	ACP	II	Anterior
104	Right	C2	C2	A2C	ACP	I	Central
105	Right	C2	C1	A2C	ACP	II	Central
202	Right	C1	C1	ACP	ACP	I	Central
203	Right	C2	C1	A2CP	ACP	I	Central
204	Right	C1	C1	A2CP	A2CP	I	Anterior
301	Right	C2	C2	A2CP	ACP	I	Central
302	Left	C2	C2	A2CP	A2CP	II	Anterior
303	Right	C2	C2	A2CP	A2CP	I	Anterior
304	Right	C2	C1	ACP	A2CP	I	Central
307	Right	C1	C1	ACP	A2CP	II	Central
309	Left	C2	C2	A2CP	A2CP	I	Central
310	Right	C2	C1	A2CP	ACP	I	Anterior
311	Left	C2	C2	A2C	ACP	I	Central
312	Right	C1	C1	A2CP	A2CP	I	Anterior
501	Left	C1	C1	ACP	ACP	II	Central
502	Left	C2	C1	A2C	ACP	I	Central
601	Left	C2	C1	ACP	ACP	I	Anterior
602	Left	C2	C2	A2CP	A2CP	II	Central
603	Left	C2	C2	A2CP	A2CP	II	Central

ON: osteonecrosis of the femoral head; WBA: Weight Bearing Area; * 2001 classification system on pre-operative T1-MR and AP X-ray, proposed by the Japanese Investigation Committee [6]; ** ACP: (from-to) Anterior, Central, Posterior. A value of 2 is added when the lesion surpasses the anterior or the posterior acetabular rim; ^†^ The WBA is divided in three equidistant areas starting from the anatomical angle (I: 1st third) to the acetabular edge (III: 3rd third).

## Data Availability

Data available on request due to restrictions e.g., privacy or ethical.
